# Comparison of physical and social risk-reducing factors for the development of disability in older adults: a population-based cohort study

**DOI:** 10.1136/jech-2019-212372

**Published:** 2019-06-26

**Authors:** Daisy Fancourt, Andrew Steptoe

**Affiliations:** Department of Behavioural Science and Health, University College London Research Department of Epidemiology and Public Health, London, UK

**Keywords:** disability, social, physical activity, cohort

## Abstract

**Background:**

Considerations of modifiable risk factors for the development of disability in older age have traditionally focused on physical activity. However, there is increasing evidence that psychological, social, and cognitive factors also help to maintain functional independence. This study compared the protective associations between physical and social activities and disability onset.

**Methods:**

We analysed data from 5434 adults aged 50+ years tracked biennially from 2004/2005 to 2016/2017, measuring self-reported difficulty in carrying out any basic activities of daily living (ADLs) or instrumental ADLs. Exposures included mild, moderate and vigorous physical activity, frequency of socialising with friends/family, cultural engagement (eg, going to the theatre/museums/concerts), and participation in community groups.

**Results:**

Over the 12-year follow-up, 1945 adults developed disability. Using Cox proportional hazards regression models adjusted for all identified demographic and health-related variables, vigorous exercise or activity once a month or more (HR 0.82, 95% CI 0.71 to 0.96), moderate exercise or activity more than once a week (HR 0.81, 95% CI 0.67 to 0.97) or cultural engagement once or twice a year or more (HR 0.84, 95% CI 0.73 to 0.97) were associated with a lower hazard of developing disability. Other exposures did not show independent protective associations. Results were robust to sensitivity analyses considering reverse causality and exploring the potential confounding role of time-invariant factors, such as socioeconomic status.

**Conclusion:**

These results suggest the importance of either developing multimodal interventions to protect against disability and promote healthy ageing or promoting greater physical and social engagement with existing community activities among older adults.

## Introduction

Disability is a major challenge among ageing populations. Indeed, it is estimated that 45% of adults aged 65 years and above experience disability.^1^ Disability is frequently categorised into limitations affecting activities essential to independent living (activities of daily living or ADLs) and desired activities important to a person’s quality of life (instrumental ADLs or IADLs).^2^ Disability can reduce autonomy and independence and also increase the risk of outpatient care, hospitalisation, nursing home admission and death.^2 3^ It is also bidirectionally associated with comorbidity and frailty.^2^ While much attention on disability research has focused on the role of core demographic factors, such as sex, education and wealth, in determining disability risk in older age,^4–7^ there is increasing research exploring potential modifiable risk factors, such as physical activity. Observational studies have identified protective associations between routine exercise and physical activity and both ADLs and IADLs.^8^ Mediators in this relationship appear to include obesity, muscle mass and muscle strength.^9 10^ Some interventional studies of both resistance and aerobic physical activity programmes have also shown promise in lowering the cumulative incidence of ADL disability compared with control groups.^11^ However, systematic reviews of interventional studies have shown mixed results.^12^ One potential reason for this is that disability is not just predicted by physical variables, so single-mode interventions may not provide complete solutions. As a result, multifactorial approaches may provide more consistent results.

There is increasing evidence that psychological, social and cognitive factors also play a role in maintaining functional independence in older adults. For example, depression has been identified as a risk factor for disability in older age,^13 14^ as has cognitive functioning,^15 16^ and both low frequency of social contact and loneliness.^17 18^ As a result, there has been a call for greater involvement of community services alongside healthcare providers and caregivers.^2 12^ Early evidence has shown associations between engagement in broad community functional activities (such as shopping), social activities (such as attending church) and physically demanding leisure activities (such as walking and gardening), and better functional maintenance in older adults.^19^ ‘Feeling useful’ to friends or family has also been found to be a risk-reducing factor, suggesting that productive community activities could be protective.^20^ Further, visiting museums has been associated with a lower risk of age-related decline.^21^ However, the potential contribution of social engagement as a risk-reducing factor for disability incidence in older age remains under-researched. Specifically, it remains unknown how the protective benefits of different types of social activities compare with better-studied physical activities. This is a critical question that underlies our ability to provide public health recommendations or to design appropriate community interventions to reduce the burden of disability. Consequently, this study sought to explore whether social activity is associated with disability incidence in adults aged 50+ years and to compare the size of association with physical activity.

## Methods

### Participants

We used data from the English Longitudinal Study of Ageing: a nationally representative cohort study of adults aged over 50 years.^22^ We used wave 2 as baseline, as this contains the richest data on social and community participation in participant questionnaires. Participants who provided data at wave 2 (2004/2005) were included and followed-up biennially until wave 8 (2016/2017), a follow-up period of 12 years. Out of 8780 core participants assessed in wave 2, 33 were excluded as they were registered blind, and 2632 already exhibited disability at baseline so were excluded. This left 6115 participants of whom 5434 provided complete data so were included in analyses.

### Measures

Disability was measured using self-report of ‘difficulty’ in independently carrying out any basic ADLs (getting in or out of bed, bathing or showering, using the toilet, dressing, eating and walking across a room) or IADLs (making telephone calls, shopping for groceries, preparing a hot meal, taking medications, doing work around the house or garden, managing money or using a map to get around in a strange place). Following previous studies, we defined disability as having difficulty carrying out one or more ADLs or IADLs (producing a binary variable).^23 24^

Three types of physical activity were measured: mild exercise or activity (including moving around at home, doing laundry and simple home repairs), moderate-intensity activity (including gardening, cleaning the car, walking at moderate pace, dancing and floor or stretching exercises) and vigorous-intensity activity (including swimming, cycling, gym work out, tennis, digging with a spade, running or aerobics).^25^ For all three, frequency was recorded as hardly ever or never, one to three times a month, once a week or more than once a week (producing a score of 0–3 with higher scores indicating higher frequency of engagement). Additionally, we devised indices for three types of social activity: frequency of socialising (face-to-face contact with friends or family), frequency of engagement with community groups (including political parties, trade unions, environmental groups, tenants/residents associations, neighbourhood watch, church or religious groups, charitable associations, evening classes, social clubs, sports clubs, exercise classes or other clubs/societies) and frequency of engagement with cultural activities (including going to museums, art galleries, exhibitions, concerts, theatre or opera). For social engagement, frequency was recoded to create a four-point scale of as less than once a year or never, once or twice a month, once or twice a week, or three or more times a week. For cultural engagement, frequency was recoded as never, less than once a year, about once or twice a year, or every few months or more. For community group engagement, participants were asked to state the number of times they had engaged in the past year, and this was divided by 12 to gather a monthly average and then categorised according to the same frequencies as cultural engagement. For all, a score of 0–3 was produced, with higher scores indicating higher frequency of engagement.

We identified factors predicting both physical and social activity and disability incidence using directed acyclic graphs.^26^ These included age (in years), gender (male or female), marital status (married/cohabiting vs single/widowed/divorced), ethnicity (white British vs other), educational qualifications (no educational qualifications, education to age 16 years/O-levels, education to age 18 years/A-levels and degree/higher qualification), total non-pension wealth (quintiles) (22), employment status (working full-time vs working part-time vs not working), eyesight (fair/poor vs excellent/very good/good), chronic pain (none/mild vs moderate/severe), depression (using the Centre for Epidemiological Studies Depression scale), frequency of alcohol consumption (1–2 days a week, 3–4 days a week, 5–6 days a week or daily), whether participants currently smoked and presence of a longstanding physical illness (including cancer, chronic obstructive pulmonary disease (COPD), diabetes, angina, arthritis or a stroke in the last 2 years).

### Statistical analysis

All analyses were carried out using Stata V.14. Cumulative incidence of disability was estimated by Kaplan-Meier method and both unadjusted and adjusted hazard ratios (HRs) of disability were calculated using Cox proportional hazards regression models to estimate HRs and 95% CIs. Survival time was measured in months from baseline (the data of the wave 2 interview) to onset of disability (measured as the date of interview at which disability was first recorded), censoring (the date of the last interview prior to drop out) or end of study (measured as the date of final interview for wave 8, 12 years later). Models were built-up sequentially to show the relative effect of different types of covariates on the association. So model 1 adjusted for demographic variables (sex, age, marital status, ethnicity, educational attainment, employment status and wealth) and model 2 additionally adjusted for health-related variables (eyesight, chronic pain, smoking and alcohol consumption). Models were all stratified by depression and presence of a chronic condition, including cancer, COPD, arthritis, stroke, diabetes and angina. Under these conditions, the proportionate hazards assumption was met (as tested using the Schoenfeld residuals test). All analyses were weighted using propensity weights to ensure national representation and to take account of differential non-response. All physical and social variables were entered simultaneously into the model so associations are mutually adjusted.

We carried out several sensitivity analyses (see supplementary tables). In order to understand whether patterns of association with disability incidence across ADLs and IADLs were comparable, we ran analyses separately for ADLs and IADLs. To help to explore reverse causality (whereby people experiencing early signs of disability had altered patterns of physical activity or social engagement), we excluded cases of disability that developed in the 2 years following baseline. In recognition that disability can be reversible, we also carried out analyses focusing specifically on long-term disability, classed as experiencing disability across 2 consecutive years or more. Given that cognition could be an additional confounding factor, we re-ran analyses adjusting for standardised scores of verbal memory, executive function, processing speed and orientation in time. In order to test for cohort effects, we also split the sample into those aged under 65 years and those aged over 65 years, and re-ran analyses. As 12.5% of participants were excluded due to missing data at random, we also conducted multiple imputations by chained equations to provide a total of 10 imputed data sets, returning the sample size to 6155. Additionally, in our main analyses, we used semi-parametric methods. But as these did not estimate the baseline hazard, we also tested whether results were consistent when using a parametric model. As the hazard function showed a monotone increasing distribution, we used Weibull proportional hazards models, with Akaike’s information criterion, Bayesian information criterion and Wald tests for ĸ=1 confirming best fit compared with other parametric proportional hazards models tested. We also incorporated interaction terms to identify the optimal configuration of physical and social engagement.

10.1136/jech-2019-212372.supp1 Supplementary data

Finally, we considered that both cultural engagement and disability are impacted by socioeconomic and educational factors. Therefore, we identified a limitation that cultural engagement could be merely a sensitivity marker of high socioeconomic status (SES) or high cognitive functioning rather than a potentially modifiable risk factor. Therefore, we ran an additional analysis using fixed effects regression. Fixed effects regression explores within-person variation with individuals serving as their own reference point, compared with themselves over time. So all time-invariant covariates (which for older adults include SES and educational attainment), are accounted for, even if unobserved.^27^ This means that any association then found between cultural engagement and disability cannot be due to these confounders. We used the same initial sample as for the main analyses but included individuals with disability at baseline (as fixed effects modelling takes into account changes over time) and modelled the time-varying nature of the social and physical exposures, covariates and disability incidence. A Hausman test confirmed the selection of a fixed effects over a random effects model and coefficients for all years were not jointly equal to zero, so time-fixed effects were included in the model.

## Results

### Participant demographics

Participants had an average age of 64.8 years (SD 8.9, range 52–99) and 52.1% were female. All participants were free from disability at baseline. However, 1945 (35.8%) developed disability over the following 12 years. Participants who went on to develop disability were on average older and a greater proportion was female, unmarried, of lower educational attainment, were not working or retired, less wealthy, had poorer eyesight, had a chronic condition, were already experiencing chronic pain and were depressed (table 1).

**Table 1:** Comparison of participant demographics for those who did and did not develop disability over a 12-year follow-up period

	Total N=5434	Disability free N=3489	Developed disability N=1945	P
Age, years (mean, SD)	64.8 (8.9)	63.1 (8.1)	68.0 (9.4)	<0.001
Gender, female (%)	2833 (52.1)	1751 (50.2)	1082 (55.6)	<0.001
Ethnicity, white (%)	5353 (98.5)	3428 (98.3)	1925 (99.0)	0.036
Marital status, coupled (%)	4029 (74.1)	2729 (78.2)	1300 (66.8)	<0.001
Educational attainment (%)				<0.001
No qualification	1991 (36.6)	1155 (33.1)	836 (43.0)
Education to age 16 years	1037 (19.1)	687 (19.7)	350 (18.0)	
Education to age 18 years	1582 (29.1)	1037 (29.7)	545 (29.0)	
Degree/further education	824 (15.2)	610 (17.5)	214 (11.0)	
Employment status (%)				<0.001
Not working/retired	3189 (58.7)	1795 (51.5)	1394 (71.7)	
Working part-time	936 (17.2)	678 (19.4)	258 (13.3)	
Working full-time	1309 (24.1)	1016 (29.1)	293 (15.1)	
Wealth (%)				<0.001
Lowest wealth quintile	712 (13.1)	375 (10.8)	337 (17.3)	
Second quintile	984 (18.1)	579 (16.6)	405 (20.8)	
Third quintile	1155 (21.3)	769 (22.0)	386 (19.9)	
Fourth quintile	1237 (22.8)	807 (23.1)	430 (22.1)	
Highest wealth quintile	1346 (24.8)	959 (27.5)	387 (19.9)	
Poor eyesight (%)	445 (8.2)	213 (6.1)	232 (11.9)	<0.001
Alcohol consumption (%)				<0.001
5 or more days a week	1373 (25.3)	918 (26.3)	455 (23.4)	
1–4 days a week	2201 (40.5)	1481 (42.5)	720 (37.0)	
Once or twice a month	655 (12.1)	424 (12.2)	231 (11.9)	
Less than once a month	1205 (22.2)	666 (19.1)	539 (27.7)	
Chronic condition (%)	1367 (25.2)	669 (19.2)	698 (35.9)	<0.001
Chronic pain (%)	158 (2.9)	69 (2.0)	89 (4.6)	<0.001
Current smoker (%)	732 (13.5)	456 (13.1)	276 (14.2)	0.25
Depressed (%)	494 (9.1)	252 (7.2)	242 (12.4)	<0.001

### Physical activity

Either moderate or vigorous exercise or activity more than once a week, or vigorous activity once a week or one to three times a month were associated with a lower hazard of developing disability over the following 12 years. There were 23% fewer cases of disability amongst individuals who engaged in vigorous activity more than once a week and 19% fewer cases amongst individuals who engaged in moderate activity more than once a week compared to amongst individuals who engaged less than once a month, when considering all identified confounding factors. However, mild exercise or activity was not significantly associated with a reduced hazard of developing disability (table 2). When considering ADLs and IADLs separately, results were consistently found across both (table 3).

**Table 2:** Adjusted HRs of disability incidence by physical and social activity

N=5434; 402 438 person-months	No (cases/censored)	Person-months	Model 1	P for trend	Model 2	P for trend
**Physical**						
Vigorous activity						
Less than once a month	1163/2855	5.5 (5.3–5.8)	1 (Ref)	<0.001	1 (Ref)	<0.001
One to three times a month	224/709	4.4 (3.9–4.9)	**0.83 (0.71–** **0.96** **)**		**0.82 (0.71–** **0.96** **)**	
Once a week	193/656	4.2 (3.7–4.7)	**0.82 (0.70–** **0.96** **)**		**0.82 (0.70–** **0.96** **)**	
More than once a week	310/1214	3.6 (3.3–4.0)	**0.76 (0.66–** **0.87** **)**		**0.77 (0.67–** **0.88** **)**	
Moderate activity						
Less than once a month	202/411	6.3 (5.7–7.0)	1 (Ref)	0.007	1 (Ref)	0.01
One to three times a month	141/355	5.4 (4.8–6.2)	0.89 (0.70–1.12)		0.89 (0.70–1.12)	
Once a week	315/816	5.3 (4.8–5.7)	0.86 (0.70–1.06)		0.88 (0.72–1.08)	
More than once a week	1234/3852	4.5 (4.3–4.7)	**0.80 (0.66–** **0.96** **)**		**0.81 (0.67–** **0.97** **)**	
Mild activity						
Less than once a month	95/210	5.9 (5.1–6.8)	1 (Ref)	0.67	1 (Ref)	0.79
One to three times a month	55/172	4.3 (3.4–5.4)	1.05 (0.73–1.50)		1.03 (0.72–1.47)	
Once a week	176/466	5.1 (4.6–5.7)	1.11 (0.84–1.46)		1.12 (0.86–1.48)	
More than once a week	1566/4586	4.7 (4.5–4.9)	1.01 (0.79–1.28)		1.02 (0.80–1.30)	
**Social**						
Cultural engagement						
Never	526/1166	5.7 (5.4–6.1)	1 (Ref)	**<** **0.** **001**	1 (Ref)	<0.001
Less than once a year	388/1049	5.1 (4.7–5.5)	0.90 (0.78–1.04)		0.92 (0.80–1.07)	
Once or twice a year	474/1501	4.4 (4.1–4.8)	**0.81 (0.71–** **0.94** **)**		**0.84 (0.73–** **0.97** **)**	
Every few months or more	504/1718	4.2 (3.9–4.5)	**0.79 (0.68–** **0.91** **)**		**0.80 (0.70–** **0.93** **)**	
Community group engagement						
Never	823/2113	5.2 (4.9–5.5)	1 (Ref)	0.27	1 (Ref)	0.47
Less than once a year	394/1278	4.3 (4.0–4.7)	**0.87 (0.76–** **0.99** **)**		0.88 (0.77–1.00)	
Once or twice a year	210/582	4.9 (4.5–5.5)	0.95 (0.80–1.11)		0.96 (0.82–1.13)	
Every few months or more	464/1461	4.5 (4.2–4.9)	0.92 (0.81–1.04)		0.93 (0.82–1.06)	
Social engagement						
Less than once a month	193/522	4.9 (4.4–5.5)	1 (Ref)	0.75	1 (Ref)	0.76
Once or twice a month	280/840	4.7 (4.3–5.2)	1.00 (0.83–1.21)		1.01 (0.84–1.22)	
Once or twice a week	857/2453	4.8 (4.6–5.1)	1.01 (0.86–1.20)		1.02 (0.97–1.21)	
Three or more times a week	562/1619	4.8 (4.5–5.1)	0.99 (0.83–1.18)		0.99 (0.83–1.18)	

Model 1 adjusted for sex, age, marital status, ethnicity, educational attainment, employment status and wealth. Model 2 additionally adjusted for eyesight, chronic pain, depression, smoking, alcohol consumption and presence of a chronic condition, including cancer, COPD, arthritis, stroke, diabetes and angina.

Bold values indicate p<0.05.

COPD, chronic obstructive pulmonary disease; Ref, reference.

**Table 3:** Adjusted HRs of disability incidence by physical and social activity specifically relating to either ADLs or IADLs

N=5434	ADLs (n=1369 failures)	P for trend	IADLs (n=1520 failures)	P for trend
**Physical**				
Vigorous activity				
Less than once a month	1 (Ref)	<0.001	1 (Ref)	<0.001
One to three times a month	**0.84 (0.70–** **1.00** **)**		0.86 (0.73–1.02)	
Once a week	**0.78 (0.65–** **0.94** **)**		**0.76 (0.63–** **0.91** **)**	
More than once a week	**0.73 (0.62–** **0.87** **)**		**0.74 (0.64–** **0.87** **)**	
Moderate activity				
Less than once a month	1 (Ref)	0.12	1 (Ref)	0.026
One to three times a month	0.92 (0.70–1.22)		0.88 (0.68–1.15)	
Once a week	0.93 (0.72–1.19)		0.89 (0.70–1.12)	
More than once a week	0.85 (0.68–1.06)		**0.81 (0.66–** **1.00** **)**	
Mild activity				
Less than once a month	1 (Ref)	0.43	1 (Ref)	0.71
One to three times a month	1.05 (0.69–1.59)		0.90 (0.60–1.34)	
Once a week	1.21 (0.86–1.69)		1.10 (0.80–1.51)	
More than once a week	1.14 (0.84–1.54)		0.97 (0.73–1.30)	
**Social**				
Cultural engagement				
Never	1 (Ref)	**0.** **021**	1 (Ref)	0.005
Less than once a year	1.01 (0.85–1.21)		0.86 (0.73–1.01)	
Once or twice a year	0.95 (0.80–1.13)		**0.78 (0.66–** **0.91** **)**	
Every few months or more	**0.83 (0.70–** **0.99** **)**		**0.81 (0.69–** **0.95** **)**	
Community group engagement				
Never	1 (Ref)	0.79	1 (Ref)	0.019
Less than once a year	**0.82 (0.71–** **0.96** **)**		0.89 (0.77–1.03)	
Once or twice a year	0.99 (0.82–1.21)		0.90 (0.75–1.07)	
Every few months or more	0.94 (0.81–1.09)		**0.86 (0.74–** **0.99** **)**	
Social engagement				
Less than once a month	1 (Ref)	0.15	1 (Ref)	0.82
Once or twice a month	1.04 (0.84–1.28)		0.87 (0.70–1.07)	
Once or twice a week	1.00 (0.82–1.21)		0.88 (0.73–1.06)	
Three or more times a week	0.92 (0.75–1.12)		0.96 (0.79–1.17)	

Adjusted for sex, age, marital status, ethnicity, educational attainment, employment status, wealth, eyesight, chronic pain, depression, smoking, alcohol consumption and presence of a chronic condition, including cancer, COPD, arthritis, stroke, diabetes and angina.

Bold values indicate p<0.05.

ADLs, activities of daily living; COPD, chronic obstructive pulmonary disease; IADLs, instrumental ADLs; Ref, reference.

Sensitivity analyses excluding those who developed disability in the 2 years reduced the association between less frequent forms of vigorous physical activity and disability and attenuated the association between moderate physical activity and disability, but maintained the finding for more frequent vigorous activity (more than once a week) (supplementary table 1). When only classifying disability as present if it lasted for more than 2 years (in order to exclude short-term physical limitations due to injury or illness), results were completely maintained (supplementary table 2). Results were also consistent when using parametric Weibull models (supplementary table 3).

### Social activity

Cultural engagement once or twice a year or more was consistently associated with a lower hazard of developing disability over the following 12 years in partially and fully adjusted models, but other forms of social activity (community group engagement and social engagement with friends and family) were not (table 2). There were 20% fewer cases of disability amongst individuals who engaged in cultural activities every few months or more compared to amongst individuals who engaged less than once a month, when considering all identified confounding factors. Sensitivity analyses showed that when considering ADLs and IADLs separately, results were found across both, but most clearly for IADLs (table 3).

When excluding those who developed disability in the 2 years following baseline did not affect findings (supplementary table 1). When only classifying disability as present if it lasted for more than 2 years, results were for cultural engagement were attenuated (supplementary table 2). When additionally adjusting for cognition, results were maintained (supplementary table 3). When splitting the sample at 65 years, associations were seen most clearly in those aged 50–65 years (supplementary table 4). Results were consistent when using imputed data sets for greater statistical power (supplementary table 5) and using parametric Weibull models (supplementary table 6). When using fixed effects models to assess whether cultural engagement was merely a proxy for higher SES, the association with ADLs was not present (fully adjusted model OR=0.85, 95% CI 0.67 to 1.07) but the association with IADLs was still present (OR=0.79, 95% CI 0.62 to 0.99) (supplementary table 7).

Overall, the inclusion of interaction terms suggested that the greater protective association was found for engaging in vigorous physical activity more than once a week while doing cultural activities once or twice a year (HR=0.62, 95% CI 0.43 to 0.90) or every few months or more (HR=0.68, 95% CI 0.48 to 0.97). Amongst individuals engaging in this latter combination of activities there were 32% fewer cases of disability compared to amongst those who engaged in both activities only infrequently.

## Discussion

This study found that moderate-vigorous activity and community cultural engagement both show protective associations with age-related disability. Specifically, vigorous activity and cultural engagement every few months or more are associated with a lower hazard of developing disability affecting both ADLs and IADLs. However, moderate physical activity and less frequent cultural engagement are only associated with a lower hazard of developing disability affecting IADLs. These analyses are observational rather than experimental, so causality cannot be assumed. However, our analyses focused only on individuals who were free from disability at baseline, and we controlled for all identified demographic, health-related and behavioural confounders. Further, our sensitivity analyses suggest that results were not merely a function of underlying unidentified physical limitations affecting physical and social behaviours as well as predisposing individuals to develop disability, and that time-invariant socioeconomic and demographic factors are not explanatory factors.

Our findings on physical activity corroborate those of previous studies finding protective associations between moderate-high-intensity physical activity and disability, but not low-intensity strengthening activities.^8 12^ Notably, previous studies have shown benefits of intensity independent of volume of exercise, with high-intensity physical activity particularly linked to better physical health functioning and lower risk of functional decline but little evidence that low-intensity has such benefits.^28 29^ Current guidelines advise at least 150 min per week of moderate exercise spread out over 5 days of 30 min each.^30^ Our findings, coupled with the previous research cited above, suggest that the intensity in this recommendation is key as moderate-intensity exercise appears sufficient to reduce the risk of developing disability, although slightly less frequent vigorous activity may have similar protective associations.

We also found comparative associations between social factors and disability. Previously, ‘social activities’ broadly have been found to be associated with better physical functioning, including entertaining, attending church and attending museums,^19^ but we identified specifically cultural engagement as being independently associated with disability. This expands on previous work showing links between visiting museums and a lower risk of decline in IADLs by highlighting the similar pattern in other cultural activities and the potential relationship with ADLs too.^21^ In hypothesising why we find these results, cultural engagement can reduce sedentary behaviours and loneliness, but as other social activities, such as socialising and community group membership, did not show such associations with disability and as low-intensity exercise also did not show protective associations, other more specific mechanisms must be at play. Cultural engagement is a cognitively stimulating activity, and higher cognitive functioning is protective against disability.^31^ Notably, we found the strongest associations of cultural engagement with IADLs, which are more cognitively driven, which would support the hypothesis that cognitive stimulation is a central mechanism. While socialising and community group membership are also cognitively stimulating, cultural engagement has been identified as a particularly strong cognitive activity that shows protective associations with both cognitive decline and the development of dementia.^32 33^ As such, given our physical activity results suggest that intensity is important, it may be that cultural engagement acts as a ‘high-intensity’ cognitive activity. Additionally, cultural engagement has been found to reduce stress,^34 35^ with stress identified as an important mediator between physical challenges, such as pain and functional limitation.^36^ While other types of social engagement could also be seen to be stress reducing, they may not be to the same extent. Indeed, community group membership is a relatively heterogeneous exposure including groups for pleasure (such as social groups) but also groups with a work-related purpose (such as trade union groups and tenants’ associations), with work activity known to be associated with stress.^37^ However, more studies are required to understand specifically why cultural engagement showed associations with disability above and beyond other types of psychosocial activities.

This study had a number of strengths, including its large, nationally representative sample size, its consistent tracking of variables biennially over a 12-year period, its use of an index of 12 different types of disability, its consistent findings across both semi-parametric and parametric survival analyses and its simultaneous comparison of both physical and social predictors of disability. However, it also had several limitations. In addition to the issues around causality discussed earlier, our results may still be affected by unidentified confounding variables. Our fixed effects modelling suggested that time-invariant confounders are not responsible for the associations presented here, but there could remain further unidentified time-varying confounders. Further, we focused on disability incidence. However, it is broadly recognised that recovery from functional impairments is possible, especially if targeted supportive steps are taken once disability begins. As such disability can be considered a dynamic condition. Future studies may, therefore, like to consider the potential of community engagement for supporting the restoration of functional independence in older adults who have become disabled.^38^ Similarly, we did not explore the competing risk of death, so future studies may like to extend the analyses presented here.

Overall, these analyses showed similar protective associations for both physical and social factors in relation to the development of disability in older age. Vigorous activity once a week or more was associated with a 23% lower hazard of developing disability, moderate activity of the same frequency was associated with a 19% lower hazard and cultural engagement every few months or more was associated with a 20% lower hazard. These results for cultural engagement suggest the importance of either developing multimodal interventions to protect against disability and promote healthy ageing, or more simply promoting greater physical and social engagement with existing community activities among older adults.

**What is already known on this subject**

- It is well known that physical activity is protective against developing disability in older age, and there is more recent evidence showing that social deficits, such as loneliness and isolation, are risk factors. But it remains unclear whether engaging in social activities could help to reduce the risk of developing disability, and how the protective association of social activities compares with better-researched physical activities is under-researched.

**What this study adds**

- This study shows independent protective associations between community cultural engagement and disability onset. Further, it shows similar sized protective associations with physical activity, especially for instrumental activities of daily living. Cultural engagement is a multimodal psychosocial activity being linked increasingly with healthy ageing. These results suggest the importance of promoting not only physical activity but also community cultural engagement among older adults to help maintain functional independence in older age.

## References

[1] Family resources survey: financial year 2016/17. GOV.UK. Available: https://www.gov.uk/government/statistics/family-resources-survey-financial-year-201617 [Accessed 1 Oct 2018].

[2] Fried LP, Ferrucci L, Darer J, et al Untangling the concepts of disability, frailty, and comorbidity: implications for improved targeting and care. J Gerontol A Biol Sci Med Sci 2004;59:M255–M263. 10.1093/gerona/59.3.M255 15031310

[3] Millán-Calenti JC, Tubío J, Pita-Fernández S, et al Prevalence of functional disability in activities of daily living (ADL), instrumental activities of daily living (IADL) and associated factors, as predictors of morbidity and mortality. Arch Gerontol Geriatr 2010;50:306–10. 10.1016/j.archger.2009.04.017 19520442

[4] Leveille SG, Penninx BW, Melzer D Sex differences in the prevalence of mobility disability in old age:the dynamics of incidence, recovery, and mortality. J Gerontol B Psychol Sci Soc Sci 2000;55:S41–S50. 10.1093/geronb/55.1.S41 10728129

[5] Grundy E, Glaser K Socio-demographic differences in the onset and progression of disability in early old age: a longitudinal study. Age Ageing 2000;29:149–57. 10.1093/ageing/29.2.149 10791450

[6] Jagger C, Matthews R, Melzer D, et al Educational differences in the dynamics of disability incidence, recovery and mortality: findings from the MRC cognitive function and Ageing Study (MRC CFAS). Int J Epidemiol 2007;36:358–65. 10.1093/ije/dyl307 17255347

[7] Makaroun LK, Brown RT, Diaz-Ramirez LG, et al Wealth-Associated disparities in death and disability in the United States and England. JAMA Intern Med 2017;177:1745–53. 10.1001/jamainternmed.2017.3903 29059279 PMC5820733

[8] Tak E, Kuiper R, Chorus A, et al Prevention of onset and progression of basic ADL disability by physical activity in community dwelling older adults: a meta-analysis. Ageing Res Rev 2013;12:329–38. 10.1016/j.arr.2012.10.001 23063488

[9] Visser M, Goodpaster BH, Kritchevsky SB, et al Muscle mass, muscle strength, and muscle fat infiltration as predictors of incident mobility limitations in Well-Functioning older persons. J Gerontol A Biol Sci Med Sci 2005;60:324–33. 10.1093/gerona/60.3.324 15860469

[10] Vincent HK, Vincent KR, Lamb KM Obesity and mobility disability in the older adult. Obes Rev 2010;11:568–79. 10.1111/j.1467-789X.2009.00703.x 20059707

[11] Penninx BW, Messier SP, Rejeski WJ, et al Physical exercise and the prevention of disability in activities of daily living in older persons with osteoarthritis. Arch Intern Med 2001;161:2309–16. 10.1001/archinte.161.19.2309 11606146

[12] Daniels R, van Rossum E, de Witte L, et al Interventions to prevent disability in frail community-dwelling elderly: a systematic review. BMC Health Serv Res 2008;8 10.1186/1472-6963-8-278 PMC2630317 19115992

[13] Kivelä SL, Pahkala K Depressive disorder as a predictor of physical disability in old age. J Am Geriatr Soc 2001;49:290–6. 10.1046/j.1532-5415.2001.4930290.x 11300240

[14] Taş U, Verhagen AP, Bierma-Zeinstra SMA, et al Incidence and risk factors of disability in the elderly: the Rotterdam study. Prev Med 2007;44:272–8. 10.1016/j.ypmed.2006.11.007 17184831

[15] Cognitive Functioning as a Predictor of Functional Disability in Later Life - The American Journal of Geriatric Psychiatry. Available: https://www.ajgponline.org/article/S1064-7481(12)61590-5/abstract[Accessed 11 Jun 2018]. 10.1097/01.JGP.0000192502.10692.d6 16407580

[16] Dodge HH, Kadowaki T, Hayakawa T, et al Cognitive impairment as a strong predictor of incident disability in specific ADL-IADL tasks among community-dwelling elders: the Azuchi study. Gerontologist 2005;45:222–30. 10.1093/geront/45.2.222 15799987

[17] Stuck AE, Walthert JM, Nikolaus T, et al Risk factors for functional status decline in community-living elderly people: a systematic literature review. Soc Sci Med 1999;48:445–69. 10.1016/S0277-9536(98)00370-0 10075171

[18] Shankar A, McMunn A, Demakakos P, et al Social isolation and loneliness: prospective associations with functional status in older adults. Health Psychol 2017;36:179–87. 10.1037/hea0000437 27786518

[19] Everard KM, Lach HW, Fisher EB, et al Relationship of activity and social support to the functional health of older adults. J Gerontol B Psychol Sci Soc Sci 2000;55:S208–S212. 10.1093/geronb/55.4.S208 11584883

[20] Gruenewald TL, Karlamangla AS, Greendale GA, et al Feelings of usefulness to others, disability, and mortality in older adults: the MacArthur study of successful aging. J Gerontol B Psychol Sci Soc Sci 2007;62:P28–P37. 10.1093/geronb/62.1.P28 17284554

[21] d'Orsi E, Xavier AJ, Steptoe A, et al Socioeconomic and lifestyle factors related to instrumental activity of daily living dynamics: results from the English longitudinal study of ageing. J Am Geriatr Soc 2014;62:1630–9. 10.1111/jgs.12990 25243677

[22] Steptoe A, Breeze E, Banks J, et al Cohort profile: the English longitudinal study of ageing. Int J Epidemiol 2013;42:1640–8. 10.1093/ije/dys168 23143611 PMC3900867

[23] Torres JL, Lima-Costa MF, Marmot M, et al Wealth and disability in later life: the English longitudinal study of ageing (ELSA). PLoS One 2016;11:e0166825 10.1371/journal.pone.0166825 27875579 PMC5119775

[24] Ormel J, Rijsdijk FV, Sullivan M, et al Temporal and reciprocal relationship between IADL/ADL disability and depressive symptoms in late life. J Gerontol B Psychol Sci Soc Sci 2002;57:P338–P347. 10.1093/geronb/57.4.P338 12084784

[25] Demakakos P, Hamer M, Stamatakis E, et al Low-intensity physical activity is associated with reduced risk of incident type 2 diabetes in older adults: evidence from the English longitudinal study of ageing. Diabetologia 2010;53:1877–85. 10.1007/s00125-010-1785-x 20495973

[26] Shrier I, Platt RW Reducing bias through directed acyclic graphs. BMC Med Res Methodol 2008;8 10.1186/1471-2288-8-70 PMC2601045 18973665

[27] Allison PD Fixed effects regression models. SAGE publications, 2009.

[28] Holstila A, Mänty M, Rahkonen O, et al Changes in leisure-time physical activity and physical and mental health functioning: a follow-up study. Scand J Med Sci Sports 2017;27:1785–92. 10.1111/sms.12758 27714910

[29] Gebel K, Ding D, Bauman AE Volume and intensity of physical activity in a large population-based cohort of middle-aged and older Australians: prospective relationships with weight gain, and physical function. Prev Med 2014;60:131–3. 10.1016/j.ypmed.2013.12.030 24398175

[30] Department of Health Physical Activity, Health Improvement and Protection, 2011. Start active, Stay active: a report on physical activity from the four home countries’ Chief Medical Officers, 2011 Available: https://www.gov.uk/government/publications/start-active-stay-active-a-report-on-physical-activity-from-the-four-home-countries-chief-medical-officers [Accessed 24 Sep 2018].

[31] McGuire LC, Ford ES, Ajani UA Cognitive functioning as a predictor of functional disability in later life. Am J Geriatr Psychiatry 2006;14:36–42. 10.1097/01.JGP.0000192502.10692.d6 16407580

[32] Fancourt D, Steptoe A Cultural engagement predicts changes in cognitive function in older adults over a 10 year period: findings from the English longitudinal study of ageing. Sci Rep 2018;8 10.1038/s41598-018-28591-8 PMC6033851 29977058

[33] Fancourt D, Steptoe A, Cadar D Cultural engagement and cognitive reserve: museum attendance and dementia incidence over a 10-year period. Br J Psychiatry 2018;213:661–3. 10.1192/bjp.2018.129 30025547 PMC6429239

[34] Fancourt D, Williamon A Attending a concert reduces glucocorticoids, progesterone and the cortisol/DHEA ratio. Public Health 2016;132:101–4. 10.1016/j.puhe.2015.12.005 26852282

[35] Camic PM, Chatterjee HJ Museums and art galleries as partners for public health interventions. Perspect Public Health 2013;133:66–71. 10.1177/1757913912468523 23308010

[36] Hall AM, Kamper SJ, Maher CG, et al Symptoms of depression and stress mediate the effect of pain on disability. Pain 2011;152:1044–51. 10.1016/j.pain.2011.01.014 21306826

[37] Kunz-Ebrecht SR, Kirschbaum C, Marmot M, et al Differences in cortisol awakening response on work days and weekends in women and men from the Whitehall II cohort. Psychoneuroendocrinology 2004;29:516–28. 10.1016/S0306-4530(03)00072-6 14749096

[38] Gill TM, Allore HG, Hardy SE, et al The dynamic nature of mobility disability in older persons. J Am Geriatr Soc 2006;54:248–54. 10.1111/j.1532-5415.2005.00586.x 16460375

