# Supplementary material for: Comparison of physical and social risk-reducing factors for the development of disability in older adults: a population-based cohort study
Source: J Epidemiol Community Health. 2019 Jun 26;73(10):906–12. doi: 10.1136/jech-2019-212372 (PMC6817696; doi:10.1136/jech-2019-212372)
Supplement: Supplementary data [file jech-2019-212372supp001.pdf]

Supplementary table 1: Adjusted hazard ratios of disability incidence by physical and social activity excluding those who developed disability in the two years following baseline

**N=4,115**	**HR (n=1,286 failures)**	**p for trend**
**PHYSICAL**		
Vigorous activity		
Less than once a month	1 [Ref]	**.028**
1-3 times a month	0.87 (0.73-1.05)	
Once a week	0.92 (0.76-1.10)	
More than once a week	**0.85 (0.72-0.99)**	
Moderate activity		
Less than once a month	1 [Ref]	.22
1-3 times a month	0.86 (0.62-1.19)	
Once a week	0.89 (0.67-1.18)	
More than once a week	0.84 (0.65-1.09)	
Mild activity		
Less than once a month	1 [Ref]	.22
1-3 times a month	1.07 (0.67-1.70)	
Once a week	1.39 (0.96-2.00)	
More than once a week	1.28 (0.92-1.77)	
**SOCIAL**		
Cultural engagement		
Never	1 [Ref]	**.001**
Less than once a year	0.86 (0.71-1.02)	
Once or twice a year	**0.78 (0.65-0.94)**	
Every few months or more	**0.74 (0.62-0.89)**	
Community group engagement		
Never	1 [Ref]	.70
Less than once a year	0.92 (0.78-1.07)	
Once or twice a year	0.99 (0.81-1.21)	
Every few months or more	0.95 (0.81-1.11)	
Social engagement		
Less than once a month	1 [Ref]	.48
Once or twice a month	0.97 (0.77-1.23)	
Once or twice a week	0.99 (0.80-1.22)	
Three or more times a week	0.94 (0.75-1.17)	

Adjusted for sex, age, marital status, ethnicity, educational attainment, occupational status, wealth, eyesight, chronic pain, depression, smoking, alcohol consumption and presence of a chronic condition including cancer, COPD, arthritis, stroke, diabetes and angina.

Supplementary table 2: Adjusted hazard ratios of long-term disability incidence (over 2 or more years) by physical and social activity

N=5,434	HR for long-term disability (n=800 failures)	p for trend
**PHYSICAL**		
Vigorous activity		
Less than once a month	1 [Ref]	**<.001**
1-3 times a month	**0.89 (0.71-1.12)**	
Once a week	**0.70 (0.54-0.90)**	
More than once a week	**0.66 (0.53-0.82)**	
Moderate activity		
Less than once a month	1 [Ref]	**.049**
1-3 times a month	1.07 (0.76-1.49)	
Once a week	0.90 (0.67-1.22)	
More than once a week	0.82 (0.63-1.08)	
Mild activity		
Less than once a month	1 [Ref]	.78
1-3 times a month	0.92 (0.55-1.53)	
Once a week	1.18 (0.77-1.79)	
More than once a week	0.97 (0.66-1.41)	
**SOCIAL**		
Cultural engagement		
Never	1 [Ref]	.24
Less than once a year	1.02 (0.82-1.28)	
Once or twice a year	1.03 (0.83-1.28)	
Every few months or more	0.97 (0.70-1.09)	
Community group engagement		
Never	1 [Ref]	.57
Less than once a year	0.81 (0.66-1.00)	
Once or twice a year	1.00 (0.78-1.29)	
Every few months or more	0.87 (0.74-1.10)	
Social engagement		
Less than once a month	1 [Ref]	.95
Once or twice a month	0.81 (0.70-1.24)	
Once or twice a week	1.00 (0.79-1.30)	
Three or more times a week	0.90 (0.76-1.29)	

Adjusted for sex, age, marital status, ethnicity, educational attainment, occupational status, wealth, eyesight, chronic pain, depression, smoking, alcohol consumption and presence of a chronic condition including cancer, COPD, arthritis, stroke, diabetes and angina.

Supplementary table 3: Adjusted hazard ratios additionally adjusted for cognition

N=5,361	HR for long-term disability (n=1,853 failures)	p for trend
**PHYSICAL**		
Vigorous activity		
Less than once a month	1 [Ref]	**<.001**
1-3 times a month	**0.82 (0.71-0.96)**	
Once a week	**0.82 (0.70-0.96)**	
More than once a week	**0.77 (0.67-0.88)**	
Moderate activity		
Less than once a month	1 [Ref]	**.016**
1-3 times a month	0.90 (0.71-1.14)	
Once a week	0.89 (0.73-1.10)	
More than once a week	**0.82 (0.68-0.98)**	
Mild activity		
Less than once a month	1 [Ref]	.92
1-3 times a month	1.06 (0.74-1.54)	
Once a week	1.15 (0.87-1.53)	
More than once a week	1.05 (0.82-1.35)	
**SOCIAL**		
Cultural engagement		
Never	1 [Ref]	**.004**
Less than once a year	0.92 (0.80-1.07)	
Once or twice a year	0.84 (0.73-0.97)	
Every few months or more	0.82 (0.71-0.95)	
Community group engagement		
Never	1 [Ref]	.61
Less than once a year	0.89 (0.78-1.02)	
Once or twice a year	0.99 (0.84-1.16)	
Every few months or more	0.94 (0.83-1.08)	
Social engagement		
Less than once a month	1 [Ref]	.83
Once or twice a month	1.03 (0.85-1.25)	
Once or twice a week	1.04 (0.88-1.23)	
Three or more times a week	1.01 (0.84-1.21)	

Adjusted for sex, age, marital status, ethnicity, educational attainment, occupational status, wealth, eyesight, chronic pain, depression, smoking, alcohol consumption and presence of a chronic condition including cancer, COPD, arthritis, stroke, diabetes and angina, and cognition (memory, executive function, processing speed and orientation in time).

Supplementary table 4: Adjusted hazard ratios split by age cohort (50-64 and 65+)

	**Age 50-64**<br>**N=2,945**		**Age 65+**<br>**N=2,404**	
	HR for long-term disability (n=757 failures)	p for trend	HR for long-term disability (n=1,134 failures)	p for trend
**PHYSICAL**				
Vigorous activity				
Less than once a month	1 [Ref]	**<.001**	1 [Ref]	**.047**
1-3 times a month	0.85 (0.67-1.07)		0.82 (0.67-1.00)	
Once a week	**0.68 (0.52-0.88)**		0.94 (0.77-1.16)	
More than once a week	**0.72 (0.59-0.89)**		0.84 (0.71-1.01)	
Moderate activity				
Less than once a month	1 [Ref]	.11	1 [Ref]	**.021**
1-3 times a month	0.86 (0.58-1.28)		0.91 (0.68-1.22)	
Once a week	1.02 (0.72-1.44)		0.81 (0.63-1.04)	
More than once a week	0.81 (0.60-1.11)		**0.78 (0.63-0.98)**	
Mild activity				
Less than once a month	1 [Ref]	.98	1 [Ref]	.90
1-3 times a month	1.20 (0.65-2.20)		0.93 (0.60-1.42)	
Once a week	1.16 (0.70-1.93)		1.10 (0.80-1.52)	
More than once a week	1.10 (0.70-1.74)		1.01 (0.76-1.33)	
**SOCIAL**				
Cultural engagement				
Never	1 [Ref]	<.001	1 [Ref]	.23
Less than once a year	0.82 (0.65-1.03)		0.96 (0.80-1.16)	
Once or twice a year	**0.71 (0.56-0.89)**		0.93 (0.78-1.11)	
Every few months or more	**0.64 (0.50-0.81)**		0.91 (0.76-1.09)	
Community group engagement				
Never	1 [Ref]	.94	1 [Ref]	.23
Less than once a year	0.96 (0.78-1.17)		**0.82 (0.70-0.97)**	
Once or twice a year	1.00 (0.76-1.30)		0.92 (0.75-1.13)	
Every few months or more	0.97 (0.78-1.20)		0.88 (0.75-1.04)	
Social engagement				
Less than once a month	1 [Ref]	.33	1 [Ref]	.27
Once or twice a month	1.04 (0.77-1.42)		1.02 (0.80-1.31)	
Once or twice a week	1.21 (0.93-1.59)		0.94 (0.75-1.17)	
Three or more times a week	1.13 (0.86-1.49)		0.94 (0.75-1.18)	

Adjusted for sex, age, marital status, ethnicity, educational attainment, occupational status, wealth, eyesight, chronic pain, depression, smoking, alcohol consumption and presence of a chronic condition including cancer, COPD, arthritis, stroke, diabetes and angina.

Supplementary table 5: Adjusted hazard ratios of disability incidence by physical and social activity using multiple imputation for missing data

**N=6,115**	**HR (n=2,166 failures)**	**p for trend**
**PHYSICAL**		
Vigorous activity		
Less than once a month	1 [Ref]	**<.001**
1-3 times a month	**0.85 (0.73-0.98)**	
Once a week	**0.81 (0.70-0.95)**	
More than once a week	**0.76 (0.67-0.87)**	
Moderate activity		
Less than once a month	1 [Ref]	**<.001**
1-3 times a month	0.84 (0.68-1.04)	
Once a week	**0.82 (0.68-0.99)**	
More than once a week	**0.74 (0.63-0.87)**	
Mild activity		
Less than once a month	1 [Ref]	.28
1-3 times a month	0.94 (0.68-1.31)	
Once a week	0.99 (0.78-1.27)	
More than once a week	0.92 (0.74-1.14)	
**SOCIAL**		
Cultural engagement		
Never	1 [Ref]	**.005**
Less than once a year	0.93 (0.81-1.08)	
Once or twice a year	**0.86 (0.75-0.99)**	
Every few months or more	**0.83 (0.71-0.86)**	
Community group engagement		
Never	1 [Ref]	.13
Less than once a year	0.87 (0.76-0.98)	
Once or twice a year	0.93 (0.79-1.09)	
Every few months or more	0.90 (0.79-1.02)	
Social engagement		
Less than once a month	1 [Ref]	.91
Once or twice a month	1.00 (0.84-1.17)	
Once or twice a week	1.03 (0.89-1.18)	
Three or more times a week	1.00 (0.86-1.16)	

Adjusted for sex, age, marital status, ethnicity, educational attainment, occupational status, wealth, eyesight, chronic pain, depression, smoking, alcohol consumption and presence of a chronic condition including cancer, COPD, arthritis, stroke, diabetes and angina.

Supplementary table 6: Adjusted hazard ratios of disability incidence by physical and social activity using Weibull survival distribution

N=5,434	HR for long-term disability (n=800 failures)	p for trend
**PHYSICAL**		
Vigorous activity		
Less than once a month	1 [Ref]	**<.001**
1-3 times a month	**0.83 (0.71-0.96)**	
Once a week	**0.82 (0.70-0.95)**	
More than once a week	**0.76 (0.67-0.87)**	
Moderate activity		
Less than once a month	1 [Ref]	**.011**
1-3 times a month	0.93 (0.74-1.16)	
Once a week	0.96 (0.79-1.17)	
More than once a week	**0.83 (0.70-0.99)**	
Mild activity		
Less than once a month	1 [Ref]	.81
1-3 times a month	0.97 (0.68-1.37)	
Once a week	1.09 (0.84-1.42)	
More than once a week	1.00 (0.79-1.25)	
**SOCIAL**		
Cultural engagement		
Never	1 [Ref]	**<.001**
Less than once a year	0.93 (0.81-1.07)	
Once or twice a year	**0.83 (0.72-0.95)**	
Every few months or more	**0.79 (0.68-0.91)**	
Community group engagement		
Never	1 [Ref]	.38
Less than once a year	0.89 (0.78-1.01)	
Once or twice a year	0.98 (0.84-1.14)	
Every few months or more	0.93 (0.82-1.05)	
Social engagement		
Less than once a month	1 [Ref]	.70
Once or twice a month	1.01 (0.84-1.22)	
Once or twice a week	1.01 (0.85-1.19)	
Three or more times a week	0.99 (0.83-1.17)	

Supplementary table 7: Fixed effects modelling predicting the association between cultural engagement and both ADLs and IADLs

	**ADLs**	**IADLs**
**Unadjusted analyses**<br>Every few months or more<br>[ref: less often]	0.88 (0.70-1.10)	**0.78 (0.62-0.97)**
Number of observations/individuals	11,598/2,084	12,186/2,189
Observations per individual (range)	5.6 (2-7)	5.6 (2-7)
**Fully-adjusted analyses**<br>Every few months or more<br>[ref: less often] Adjusted	0.85 (0.67-1.07)	**0.79 (0.62-0.99)**
Number of observations/individuals	10,004/1,893	10,622/2,016
Observations per individual (range)	5.3 (2-7)	5.3 (2-7)

The model automatically takes into account all time-invariant factors such as sex, age, ethnicity, socio-economic status and educational attainment. Adjusted models also include time-varying marital status, employment status, wealth, eyesight, chronic pain, depression, smoking, alcohol consumption, presence of a chronic condition including cancer, COPD, arthritis, stroke, diabetes and angina, social engagement, community group engagement and physical activity.
